# Unraveling the Photocatalytic Mechanisms on TiO_2_ Surfaces Using the Oxygen-18 Isotopic Label Technique

**DOI:** 10.3390/molecules191016291

**Published:** 2014-10-10

**Authors:** Xibin Pang, Chuncheng Chen, Hongwei Ji, Yanke Che, Wanhong Ma, Jincai Zhao

**Affiliations:** Beijing National Laboratory for Molecular Sciences, Key Laboratory of Photochemistry, Institute of Chemistry, The Chinese Academy of Sciences, Beijing 100190, China

**Keywords:** TiO_2_ photocatalysis, oxygen isotope, hydroxylation, ring-opening, decarboxylation

## Abstract

During the last several decades TiO_2_ photocatalytic oxidation using the molecular oxygen in air has emerged as a promising method for the degradation of recalcitrant organic pollutants and selective transformations of valuable organic chemicals. Despite extensive studies, the mechanisms of these photocatalytic reactions are still poorly understood due to their complexity. In this review, we will highlight how the oxygen-18 isotope labeling technique can be a powerful tool to elucidate complicated photocatalytic mechanisms taking place on the TiO_2_ surface. To this end, the application of the oxygen-18 isotopic-labeling method to three representative photocatalytic reactions is discussed: (1) the photocatalytic hydroxylation of aromatics; (2) oxidative cleavage of aryl rings on the TiO_2_ surface; and (3) photocatalytic decarboxylation of saturated carboxylic acids. The results show that the oxygen atoms of molecular oxygen can incorporate into the corresponding products in aqueous solution in all three of these reactions, but the detailed incorporation pathways are completely different in each case. For the hydroxylation process, the O atom in O_2_ is shown to be incorporated through activation of O_2_ by conduction band electrons. In the cleavage of aryl rings, O atoms are inserted into the aryl ring through the site-dependent coordination of reactants on the TiO_2_ surface. A new pathway for the decarboxylation of saturated carboxylic acids with pyruvic acid as an intermediate is identified, and the O_2_ is incorporated into the products through the further oxidation of pyruvic acid by active species from the activation of O_2_ by conduction band electrons.

## 1. Introduction

Heterogeneous photocatalytic oxidation (HPO) based on TiO_2_ as photocatalyst and solar light has emerged as a promising route for the degradation of persistent organic pollutants [1,2,3]. During photocatalytic oxidation, illumination of TiO_2_ with light energy larger than the band gap generates conduction band electrons (e^−^_cb_) and valence band holes (h^+^_vb_), which are the initial “reactive reagents” of TiO_2_ photocatalysis. The h^+^_vb_ and e^−^_cb_ can react with the H_2_O solvent and the dissolved molecular oxygen, respectively, to produce various reactive oxygen species, such as hydroxyl radicals (∙OH), hydrogen peroxide (H_2_O_2_), superoxide radicals (O_2_∙^−^) or hydroperoxyl radicals (∙OOH) [4,5]. However, the roles of H_2_O and O_2_, and the details of their photocatalytic reaction pathways are still elusive. Generally, the degradation reaction of organic pollutants is believed to be initiated by the ∙OH radical, which is formed through the oxidation of H_2_O by h^+^_vb_ and can oxidize almost all organic compounds [6]. O_2_, the final oxidant of the whole photocatalytic oxidation process was exclusively considered as a scavenger of e^−^_cb_ to depress the recombination of photogenerated h^+^_vb_/e^−^_cb_ pairs and regenerate the photocatalyst. According to this mechanism, the trapping of the e^−^_cb_ by O_2_ will only determine the rate of the photocatalytic reaction, but not change the reaction pathway and mechanism. However, recent studies have indicated that the participation of O_2_ in the photocatalytic reaction would greatly influence the degradation product distribution. For example, in the photoelectrochemical degradation of 4-chlorophenol, when the e^−^_cb_ is removed by using an appropriate bias, instead of by dioxygen [7], the mineralization of 4-chlorophenol cannot occur any more. This observation suggests that molecular oxygen may play an important role in the degradation mechanism such as the opening of aromatic rings and the subsequent mineralization, and not just act as an electron acceptor. Nevertheless, detailed mechanisms for the roles of molecular oxygen in photocatalysis are not fully understood so far.

One important reason that hinders the understanding of the mechanisms is the complexity of the photocatalytic process. In the photocatalytic reaction, the h^+^_vb_-induced oxidation half reaction and the e^−^_cb_-induced reduction half reaction proceed on the surface of one photocatalyst particle (usually of a nano size) at the same time, which makes it difficult to distinguish them in space and time. Moreover, the photocatalytic reaction involves a series of active free radical species and processes. It is challenging to investigate these species and processes with steady-state techniques. The isotopic labeling method is one of the most powerful techniques to unravel complicated reaction mechanisms [8]. Stable isotope marking, especially by ^13^C/^12^C, H/D (D = ^2^H) and ^18^O/^16^O, is a versatile analytical tool across many realms of science [9]. In the TiO_2_ photocatalytic system, the main reaction components O_2_, H_2_O and TiO_2_ all contain oxygen atoms. Accordingly, oxygen atom isotopic labeling can be the most direct and reliable method to trace the O-atom origin of products and distinguish the role and pathways of these components in the different photocatalytic reactions. Another advantage of oxygen isotopic labeling technique is its flexibility, *i.e.*, each component such as ^18^O-labeled ^18^O_2_ [10,11,12,13,14], H_2_^18^O [15,16,17], Ti^18^O_2_ [18,19,20,21,22,23] and ^18^O-labeled substrate [24,25,26] can be labeled.

On the TiO_2_ surface, the ^18^O-labeled method has been frequently used in oxygen isotopic exchange measurements to study the stability of surface oxygen in thermally activated catalytic reactions [27,28]. More often, this method was employed in gas phase TiO_2_ photocatalytic systems to investigate the photoinduced oxygen isotopic exchange with the aim of understanding the evolution of the intermediate species on the TiO_2_ surface [29,30,31,32,33,34,35,36]. However, the photoinduced oxygen isotopic exchange and oxygen isotopic exchange on TiO_2_ surface hinder the application of the isotopic labeling method in aerated aqueous TiO_2_ photocatalysis systems, because the isotope scrambling among reaction components can make the assignment of the origin of the intermediates and products uncertain. On the other hand, many researchers have also reported that, in the gas phase, the adsorption of water and organic species would inhibit the progress of the photoinduced oxygen isotopic exchange [37,38,39,40]. All this suggests that, by deliberate selection of the appropriate photocatalytic systems and conditions, the photoinduced oxygen isotopic exchange can be largely avoided, even if the reaction is carried out in aerated aqueous solutions. In fact, the oxygen isotopic exchange and photoinduced oxygen isotopic exchange between the O_2_ and TiO_2_ or between O_2_ and H_2_O were shown to be rather slow in the aqueous TiO_2_ photocatalysis systems, compared to the photocatalytic oxidation reaction in the gas phase [41,42,43,44,45]. Accordingly, in many situations, ^18^O-labeled methods can be employed to trace the pathway of oxygen-involved reaction in aqueous photocatalytic systems.

**Scheme 1:**
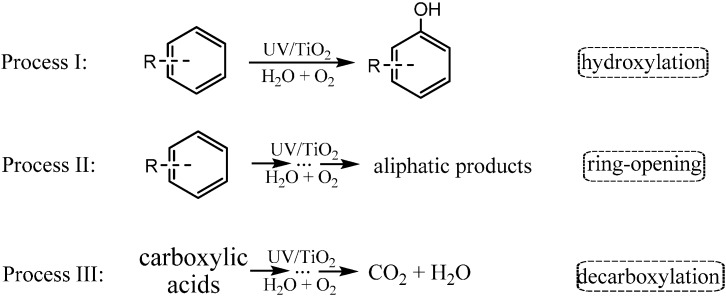
The three main processes of the TiO_2_ photocatalytic oxidation of aromatic compounds.

Aromatic rings are basic constituents of many kinds of organic pollutants, such as dyes, explosives, pesticides and pharmaceuticals. The release of these compounds could greatly affect the environment and human health [46,47]. Accordingly, aromatic compounds are the most frequently used model substrates to investigate photocatalytic mechanisms and to test the activity of photocatalysts. Before the complete mineralization of the aromatic compounds into CO_2_ and H_2_O, the photocatalytic degradation would proceed through many main intermediates with different functional groups. For example, hydroxylation, in which the hydrogen on the aromatics is replaced by the electron-donating hydroxyl (Scheme 1 process I), is regarded as an important process in the degradation of aromatic contaminants, especially at the beginning stage of the reaction [6,48,49,50,51]. On the way of mineralization, the cleavage of the aromatic ring, sometimes the hydroxylated one, to aliphatic compounds represents another critical process (Scheme 1 process II). The most stable intermediates after cleavage of aromatic ring should be the aliphatic carboxylates. The oxidative decarboxylation of these intermediates would lead to the formation of CO_2_ and H_2_O (Scheme 1 process III). Accordingly, this review is organized along these three processes: (1) hydroxylation of aromatics; (2) oxidative cleavage of aromatic rings; and (3) decarboxylation of saturated carboxylic acids, and tries to shed light on the application of ^18^O-labeling methods to the study of photocatalytic mechanisms. We will first introduce how to distinguish the O-source and the investigation of the O_2_-incorporation mechanism in the photocatalytic hydroxylation of aromatics by ^18^O-labeling methods [41,52]. The application of ^18^O-labeling methods in the study of the TiO_2_-photocatalytic aryl ring-opening mechanism is illustrated by photocatalytic degradation of 3,5-di-*tert*-butylcatechol (DTBC) in aqueous solution [53]. Finally, we will focus on ^18^O-labeling studies of the decarboxylation pathways of saturated mono- [44] and dicarboxylic acids [43]. These studies revealed that molecular oxygen can incorporate into the products to a different extent during all three of these processes, which means that O_2_ is not just a conduction band electron scavenger, but also plays a crucial role in the degradation and mineralization of organic pollutants. Moreover, we also give a detailed picture of how molecular oxygen incorporates into the products. These studies also demonstrate that ^18^O-isotope labeling is a very reliable and powerful method to study aspects of the mechanism of photocataytic oxidations, such as the role and reaction pathway of molecular oxygen and the solvent (water) in the photocatalytic reaction.

## 2. TiO_2_ Photocatalytic Hydroxylation of Aromatics

**Scheme 2:**
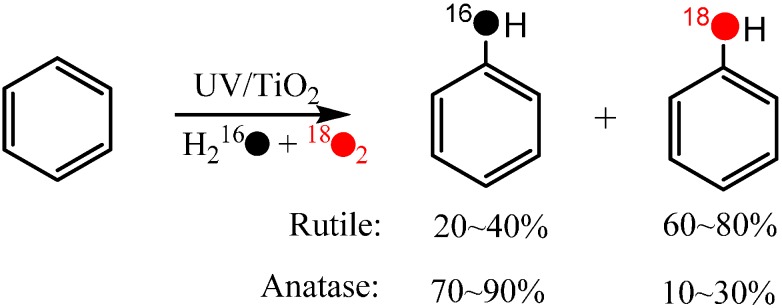
TiO_2_ photocatalytic oxidation of benzene to phenolin aerated aqueous solution [51].

During the TiO_2_ photocatalytic degradation of aromatic compounds, hydroxylation products are always the main detected intermediates. It is also accepted that the hydroxylation process is the primary one, and sometimes even the rate-determining step of the whole photocatalytic degradation reaction of aromatic compounds. Usually, the hydroxylation of aromatics is believed to be initiated by direct oxidation by h_vb_^+^ followed by hydrolysis or the attack of ·OH (formed from the oxidation of water by h_vb_^+^) in the photocatalytic systems. According to both pathways, the O-atom of the hydroxyl group in the hydroxylated product should come from H_2_O. However, while using the ^18^O-isotope labeling method (H_2_^18^O and ^18^O_2_) to investigate the process of photocatalytic oxidation of benzene to CO_2_ in aqueous solution, Matsumura and coworkers [51] found that the oxygen atoms of molecular oxygen were introduced into the phenol hydroxylation products (Scheme 2).

Moreover, the incorporation of O atoms from O_2_ exhibited great differences between the anatase and rutile systems, *i.e.*, 10%–30% and 60%–80% of the O atoms in the phenol are from O_2_ in the anatase and rutile systems, respectively. This observation has at least two implications: (1) dioxygen can participate directly in the oxidation process and incorporate it’s O-atom into the hydroxylation products; (2) the extent of the O_2_-incorporation is dependent on the photocatalytic conditions, such the crystal phase of the photocatalyst.

**Scheme 3:**
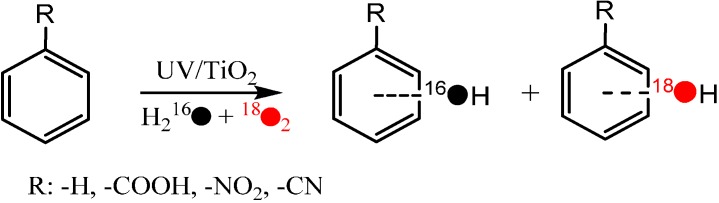
The photocatalytic hydroxylation of benzene derivatives on TiO_2_ (P25) in aerated aqueous solution [41].

In order to unravel the detailed pathway of oxygen incorporation from O_2_ in photocatalytic hydroxylation of aromatic compounds, the photocatalytic hydroxylation of several model substrates, such as benzoic acid, benzene, nitrobenzene, and benzonitrile were investigated by the isotope labeling method to trace the origin of the O atoms (from oxidant O_2_ or solvent H_2_O) in the hydroxyl groups of their corresponding hydroxylation products (Scheme 3) [41]. The results showed that, as reported by Matsumura *et al.*, the O atoms in the hydroxyl groups of the hydroxylation products can originate from both H_2_O and O_2_, and their contributions are comparable to each other in the hydroxylation process. More importantly, the percentage of hydroxylation products from O_2_ was found to depend markedly on the reaction conditions [41], such as the irradiation time and substrate concentration. The percentage of O_2_-incorporation in hydroxybenzoic acid (BA-OH), for example, increased from 33.0% to 40.1% as the irradiation time increased from 1 h to 2 h at an initial benzoic acid concentration of 3 mM. In addition, when the initial concentration of benzoic acid increased from 3 mM to 25 mM, the percentage of O_2_-incorporation in BA-OH decreased from 33.0% to 13.4% for the same irradiation time. Such a dependence of the percentage of O_2_-incorporation in hydroxylation products on the irradiation time and initial substrate concentration was also observed in the photocatalytic hydroxylation of nitrobenzene and benzonitrile.

It was also observed that when benzoic acid and benzene, which have different adsorption abilities on TiO_2_, coexisted in the same reaction system, the percentage of O_2_-incorporation in their hydroxylation products was different [41]. The addition of benzene to benzoic acid lowered slightly the percentage of O_2_-incorporation in BA-OH, while the addition of benzoic acid in benzene notably increased the percentage of O_2_-incorporation into its phenol hydroxylation product. The condition-dependence of the percentage of O_2_-incorporation provides an excellent opportunity for us to probe further into the role and the mechanism of O_2_ in the photocatalytic reaction.

**Scheme 4:**
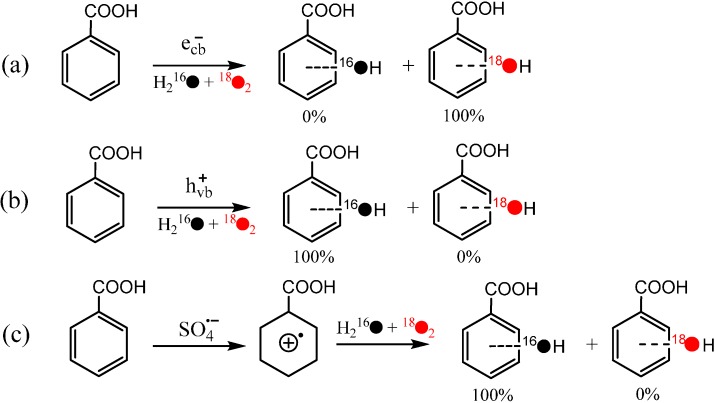
(**a**) The hydroxlyation of benzoic acid initiated by e^−^_cb_; (**b**) the hydroxlylation of benzoic acid initiated by h^+^_vb_; (**c**) the hydroxlylation of benzoic acid via the path of one-electron oxidation by SO_4_^∙^^−^ [41].

Further, by selective removal of the reactive species generated from the water oxidation by h^+^_vb_ or from O_2_ reduction by e^−^_cb_, the effect of every reactive species on the isotope distribution of the hydroxylated product was investigated systematically. When formic acid was used to selectively remove the h^+^_vb_, the hydroxylation reaction still occurred. Moreover, nearly all O atoms in the hydroxyl groups of the hydroxylated products of benzoic acid came from O_2_ in this situation (Scheme 4a). On the other hand, by employing the benzoquinone to scavenge e^−^_cb_ or oxidize O_2_^−^ back to O_2_, the hydroxyl O atoms almost all originated from the H_2_O solvent (Scheme 4b). Such a sharp contrast in the isotope distribution indicates that h^+^_vb_ is indispensable to H_2_O incorporation and H_2_O cannot participate in the hydroxylation of aromatic compounds if the oxidation of H_2_O by h^+^_vb_ is blocked, while e^−^_cb_ is indispensable to O_2_ incorporation. These observations do not support the earlier proposed mechanism that the direct reaction between O_2_ and the substrate radical species formed by hole oxidation or HO^∙^-adduct is the main O_2_-incorporation pathway in the hydroxylation process, but imply that the O_2_ has to be activated by e^−^_cb_ before its incorporation. This argument is further confirmed by the isotope experiment of directly oxidation benzoic acid to its cation radical by the one-electron oxidant SO_4_^∙−^, in which the hydroxyl O atoms were observed to nearly all from H_2_O (Scheme 4c).

In addition, the accumulation of H_2_O_2_ was observed to be dependent on the concentration of substrates. The higher concentration of benzoic acid led to the slower consumption of H_2_O_2_, which is attributed to the competitive adsorption on TiO_2_ between H_2_O_2_ and benzoic acid. The different percentages of O_2_-incorporation in their hydroxylation products between benzoic acid and benzene, which have different adsorption abilities on TiO_2_, also demonstrate that the adsorption is a key factor that determines the O_2_-incorporation. All the experimental results indicate that the reaction on the surface tends to incorporate the more O atoms from H_2_O into the product, while the hydroxylation of the unabsorbed substrate leads to the formation of the product containing the more O_2_-derived O atoms. Therefore, both the pathways of the direct oxidation by h^+^_vb_ with further hydrolysis (only from H_2_O) on the surface and the addition of ∙OH (from both H_2_O and O_2_) in bulk solution are important in the TiO_2_ photocatalytic hydroxylation of aromatics.

**Scheme 5:**
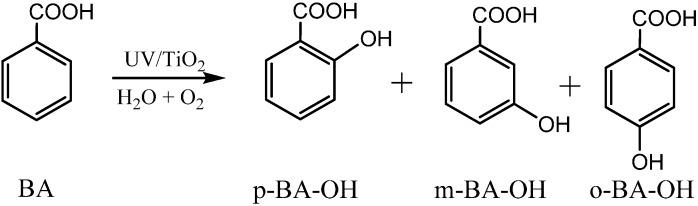
The formation of the three regioisomeric monohydroxylated products of benzoic acid: *meta*-hydroxyl benzoic acid (*m*-BA-OH), *para*-hydroxyl benzoic acid (*p*-BA-OH), and *ortho*- hydroxyl benzoic acid (*o*-BA-OH) [52].

For substituted aromatic rings, different regioisomeric hydroxylated products, which usually exhibit different biological toxicity and secondary reactivity, can be formed during the photocatalytic hydroxylation. By detailed analysis of the monohydroxylated products of benzoic acid, it was found that three isomers of BA-OH, *i.e.*, *meta*-(*m*-), *para*- (*p*-), and *ortho*- (*o*-) BA-OH, were all formed during the phtotocatalytic hydroxylation reaction (Scheme 5) [52]. The isotopic labeling experiments to trace the change of the oxygen source of the formed isomeric hydroxylated intermediates showed that the proportions of oxygen atom of the three hydroxylated isomers were remarkably different. The proportion of O atom of *m*-BA-OH from H_2_O was higher than those of *p*- and *o*-BA-OHs. These observations are somewhat unexpected, because the hydroxylation reaction should have the same oxygen source if the same active species and hydroxylated mechanism are responsible for the hydroxylation in the same photocatalytic system. In addition, the difference in the isotopic abundance of product isomers increased stably with the decrease of partial pressure of O_2_ (P_O2_) and with the increase of substrate concentrations. The analysis of the monohydroxylated products of benzoic acid indicated that three isomers of BA-OH have different yields, and that the yield distributions of these three monohydroxylated products changed with P_O2_ and substrate concentration. The formation of *m*-BA-OH was depressed relative to the *p*- and *o*-BA-OH with the increase of P_O2_, while the high substrate concentration favored the formation of *m*-BA-OH and disfavored the formation of *p*- and *o*-BA-OH, which is consistent with the changes in the isotope abundance.

**Scheme 6:**
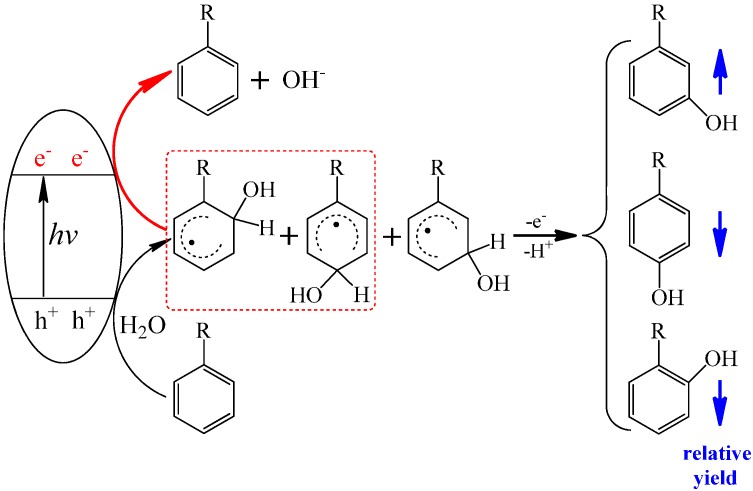
The selective reduction of HO-adduct radicals by conduction band electrons (e^−^_cb_) back to the original substrate [52].

The theoretical calculation indicated that the standard reduction potentials (E° *vs.* NHE) of the HO-adduct benzoic acid radicals at *p*- and *o*-positions are −0.22 and −0.27 V, respectively, while *m*-HO-BA radical has an E° of −0.66 V. The redox potentials of formed *p*- and *o*-BA-OH adduct radicals are below the bottom of conduction band of TiO_2_ (−0.29 V), and these adduct radicals can be easily reduced by e^−^_cb_. In contrast, the reduction of *m*-BA-OH radical by e^−^_cb_ is impossible because of the more negative reduction potential of *m*-BA-OH adduct radical. Confirming this observation, the redistribution of electron density in the presence of extra e^−^_cb_ indicates that TiO_2_ with adsorbed HO-adduct radical in the the *p*- and *o*- positions of benzoic acid, the added electron distributes mainly on the HO-BA radical, whereas this electron spreads predominantly over the d-orbits of Ti-atoms, which make up the conduction band of TiO_2_, when *m*-HO-BA radical is adsorbed on the TiO_2_ cluster [52]. Evidently, the reduction of *m*-BA-OH radical by e^−^_cb_ occurs on the surface of TiO_2_, where the O atoms of hydroxylated products predominantly come from H_2_O. However, in the bulk solution where no reductive e^−^_cb_ is available, all three HO-adduct radicals would transform into the corresponding hydroxylated products. Accordingly, any factor that can influence the adsorption of substrates and the accumulation of e^−^_cb_ would change the yield distribution and O-origin of three isomeric hydroxylated products. For example, the lower P_O2_ and higher substrate concentration all would exaggerate the accumulation of e^−^_cb,_ which favors the reduction of *p*- and *o*-BA-OH radicals. As a result, the relative ratio of *m*-BA-OH will increase. The concept that the e^−^_cb_ can selectively recombine the formed surface HO-adduct radicals back to the original substrate (Scheme 6) implicates that we can modulate the hydroxylated intermediates distribution in the photocatalytic degradation of aromatic pollutants by tuning the Fermi level of the photocatalyst or controlling the accumulation amount of e^−^_cb_ or surface modification.

Another example for the isotope-labeling study on photocatalytic mechanism comes from the modulation of O_2_ reduction pathway by pendant proton relay [42]. In the photocatalytic oxidation of benzoic acid and benzene, it was found that the O_2_-incorporation in the hydroxylation products was markedly depressed by the addition of phosphate, which can adsorb strongly on the surface of TiO_2_. Further, the isotope-labeling analysis (H_2_^16^O and ^18^O_2_) indicated that nearly 50% of the O-atoms of hydroxyl groups in BA-OH were derived from O_2_ for pristine TiO_2_, while almost no O-atoms from O_2_ were detected for the phosphate system with 2 mM phosphates. Similarly, for the photocatalytic oxidation of the weak-adsorbed benzene the significantly decreased O-atom incorporation from O_2_ into phenol was also observed upon addition of phosphate (from about 50% to 10%) [42]. Since the O_2_-incorporation is attributed to the sequential reduction of O_2_ by e^−^_cb_, as mentioned above, the depression in O_2_-incorporation is an indication of new reduction pathway of O_2_ by e^−^_cb_ in the presence of phosphates. Indeed, a detailed examination on the formation and decomposition of H_2_O_2_ during photoctalytic oxidation showed that the little H_2_O_2_ was generated in the presence of phosphates, while plenty of H_2_O_2_ was accumulated in the pristine TiO_2_ system. The following electrochemical experiments confirmed that O_2_ in the phosphate systems is reduced to H_2_O via direct four electrons reduction (Equation (1)), while the pathway of the sequential single electron reduction to H_2_O_2_ (Equations (2–5)) is bypassed. Such a change in the O_2_ reduction pathway is proposed to result from the management of surface proton by formation of “pendant proton relay structure” in which the surface-adsorbed phosphate has a pendant acid/base site [42]:

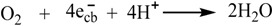
(1)

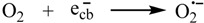
(2)

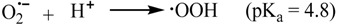
(3)

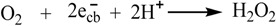
(4)

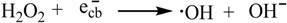
(5)

## 3. Photocatalytic Cleavage of Aryl-Ring on TiO_2_ Surface

**Scheme 7:**
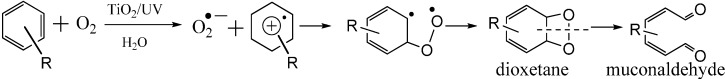
The previously proposed mechanism for TiO_2_ photocatalytic cleavage of aryl-ring via a dioxetane intermediate.

Another essential step on the way to the complete mineralization of aromatic pollutants into CO_2_ is ring-opening, which involves the C-C bond cleavage of aryl rings and is more complex than the hydroxylation process. Accordingly, our understanding on the mechanism of cleavage of aryl ring is even poorer. Many researchers still believe that the ∙OH radicals are the main active oxygen species in the ring-opening step due to their high oxidative ability [6]. However, recent electrochemical studies suggested that the aromatic rings cannot be efficiently cleaved by the ∙OH radicals in the absence of O_2_ [7], indicating the ∙OH radicals are not a good active species for the cleavage of aryl rings. Other reports [54,55,56,57,58,59] also argued that the reaction of the O_2_-derived species, such as superoxide or singlet oxygen, with aromatics is the main pathway in photocatalytic ring-opening step. It is also considered that both the ∙OH radicals and O_2_ are both important in the aromatic ring cleavage, *i.e.*, ∙OH radicals attack the aromatic ring, and the cleavage results from the reaction of the radicals formed by OH-attack and O_2_ [55,60]. As a matter of fact, whether ∙OH radical or O_2_ ultimately breaks the C-C bond of aromatic ring is still experimentally under debate and the mechanism of the ring-opening reaction in most reports remains somewhat speculative. Another popular mechanism in the ring-opening step for TiO_2_ photocatalysis is that the superoxide radical anion, generated from the reduction of O_2_ by e_cb_^−^, reacts with substrate radical cation which is formed from oxidation of the substrate by h_vb_^+^, to form a dioxetane intermediate, then homolytic cleavage of the C-C bond leads to the formation the muconaldehyde (Scheme 7) [33,55,58,61,62,63,64].

However, no direct evidence was provided so far to support this proposal. In aqueous solution, the detected initial ring-opening products are always carboxylic acids or carboxylic acid derivatives, instead of the expected dialdehyde, probably because the aldehyde is an unstable intermediate and it can be rapidly converted into a stable carboxylic acid under photocatalytic conditions. Another possibility for the low yield of the aldehyde was attributed to the hydroxylation of the aromatic ring before the cleavage, while the ring cleavage still occurs via a dioxetane intermediate process. Recently, Matsumura *et al.* [65] indeed detected the muconaldehyde intermediate during the cleavage of benzene rings by TiO_2_ photocatalyst although its yield is also very low in aqueous solution. Unfortunately, they could not track the origin of O atoms introduced into the muconaldehyde due to the fast oxygen exchange between carbonyl groups of the muconaldehyde and water. In order to track the ring-open pathway by avoiding the oxygen exchange between the products and the solvent, the *ortho*-dihydroxybenzenes were used as model substrates, because the oxidative cleavage of *ortho*-dihydroxy-benzenes would form directly the muconic acid which cannot exchange its oxygen atom with H_2_O. Therefore, the behaviours of oxygen in the ring-opening products can be definitely tracked by the ^18^O-labeling methods in the aqueous TiO_2_ photocatalytic system. Further, among the numerous *ortho*-dihydroxyl substituted benzenes, 3,5-di-*tert*-butylcatechol (DTBC) is an ideal molecular probe for the ring-cleavage, because its substituents can distinguish the ring-opening position and the steric effect of bulky *t*-butyl groups also can induce the primary ring-opening reaction with unexpectedly high yields [53]. Moreover, the pathway for oxidative cleavage of aromatic ring of DTBC has been extensively studied in the systems of catechol oxygenases and its artificially synthesised iron-containing analogues.

**Scheme 8:**
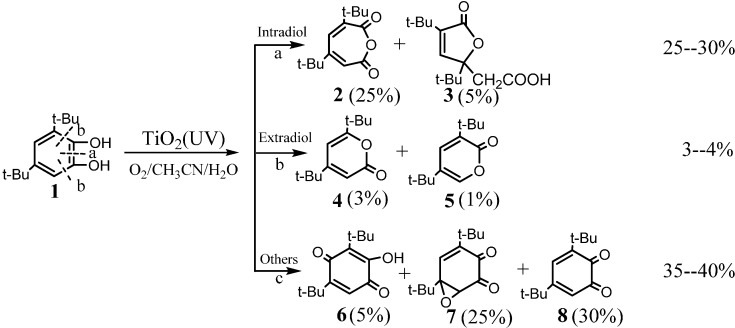
The structures of the main intermediate products of photocatalytic cleavage of 3,5-di-tert-butylcatechol (DTBC) by TiO_2_ (P25) in aerated, water/acetonitrile mixed solution. The main products are divided into three groups and the values in brackets indicate the highest yields of the corresponding products [53].

As illustrated in Scheme 8, the main oxidation products are generally divided into three groups: (1) products **2** and **3** are the primary ring-opening products without the loss of a carbon atom, and they are generally intradiol products, which means that the O-atom was inserted into the C-C bond between the two *ortho* hydroxyls; (2) products **4** and **5** have a loss of one carbon relative to their matrix and are generally extradiol products because the O-atom is inserted into the C-C bond out of the two hydroxyls; and (3) products **6**–**8** are the products by simple oxidation of the DTBC in which no aromatic C-C bond is completely broken. The yields of these products are known to change over a wide range, dependent on the reaction conditions, oxidation systems and reaction mechanisms.

Under the optimized reaction conditions [53], the total yield of the identified products during photocatalytic oxidation of DTBC could account for nearly 75% of the initial DTBC, and the initial ring-opening products (**2**+**3**+**4**+**5**) had a yield of 30% (Scheme 8), which means that most of the products were recovered after the photocatalytic reaction. In addition, the isotope exchange experiment of the initial ring-opening products **2**–**5** with H_2_^18^O or ^18^O_2_ showed that the oxygen isotopic exchange or the photoinduced oxygen isotopic exchange process was slow and can be ignored under the present experimental conditions. All these characteristics of the oxidation products of DTBC make it possible to conveniently determine the oxygen sources and accurately quantify the ring-opening products by oxygen-18 isotope labeling experiments. Among these products, products **2** and **3** are the intermediates that most directly reflect the aromatic ring-opening mechanism. The product **2** had the highest yield among all of the cleavage products (~25%). In addition, it has been widely accepted in the literature and confirmed by our experiments that product **2** is the precursor of product **3**, through the hydrolysis and subsequently addition reactions. Therefore, product **2** bears the direct information of the primary ring-opening process and is the most desirable intermediates to trace the oxygen source by isotope method.

**Figure 1:**
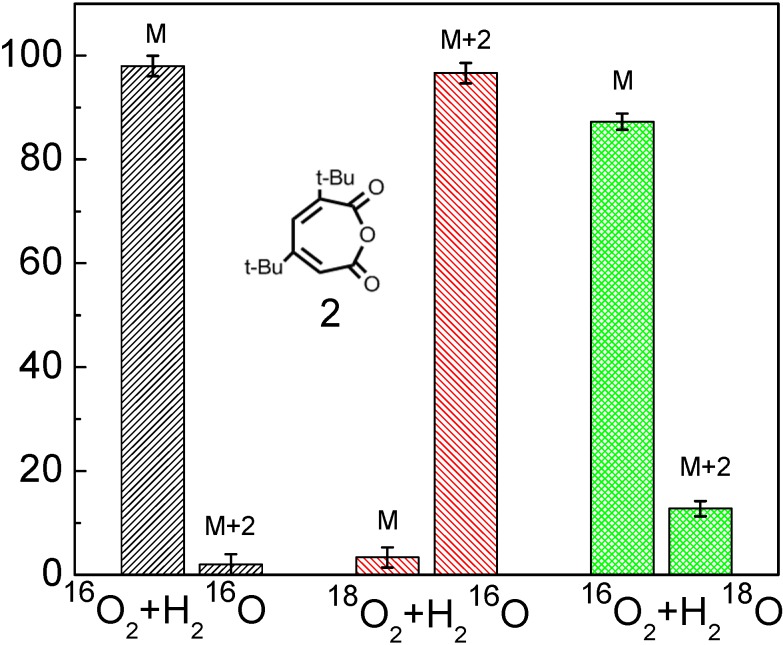
The oxygen-isotope distribution of product **2** under the various isotope conditions. In each panel, the horizontal axis represents the three isotope conditions: (1) Natural ^16^O_2_ and H_2_^16^O; (2) ^18^O_2_ and H_2_^16^O; (3) ^16^O_2_ and H_2_^18^O; the vertical axis represents the oxygen-isotope distribution ratio (%); M, M+2, M+4 denote products including 0, 1, 2 atoms of ^18^O in place of ^16^O [53].

The isotope-labeling result showed that the inserted O atom (the bridging oxygen in anhydride functional group) in the ring of product **2** was from the O_2_ in both ^18^O_2_/H_2_^16^O and ^16^O_2_/H_2_^18^O systems (Figure 1), which provides the most direct information on how the O_2_ cleaves the aromatic ring in the photocatalytic reaction [53].

**Scheme 9:**
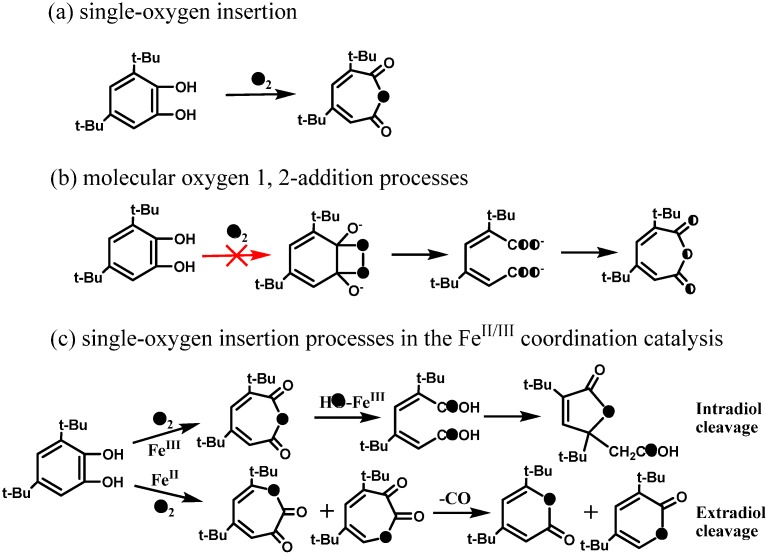
The oxygen-isotope distribution of product **2** via the (**a**) single oxygen insertion process or (**b**) molecular oxygen 1,2-addition process. (**c**) the single oxygen incorporationprocesses in biological systems [53].

This observation seems not to support the earlier proposal of the usual ring opening pathway via a dioxetane intermediate (Scheme 7). If the TiO_2_ photocleavage of aromatics is through the hemolysis of dioxetane intermediate, the major ring-opening products should be a diacid (muconic acid) or acid-aldehydes. However, in the primary ring-opening products, only product **2** was detected in the photocatalytic reaction. No evidence for muconic acid formation could be obtained, neither by HPLC-ESI nor GC-MS detection of the compound itself or of its silylated derivatives. Two possibilities may explain this observation: muconic acid is formed but rapidly converts to product **2**, or muconic acid is not formed at all and product **2** is directly derived from the insertion of an O atom in the C-C bond of DTBC. The former can be excluded because only one O atom was labeled in product **2** (Scheme 9a). If the product **2** were derived from lactonization of muconic acid, it is impossible for product **2** to be completely preserved with one labeled O atom, because the two oxygen atoms in the carboxylate anions are equal (Scheme 9b). Therefore, the isotopic labeling experiments indicate that the inserting O-atom of O_2_ into the C-C bond can lead to the cleavage of the aromatic rings in photocatalytic systems.

This single oxygen insertion from O_2_ was also found to be dominant in the formation of products **4** and **5** by using ^16^O_2_/H_2_^18^O or ^18^O_2_/H_2_^16^O. These results imply that the O_2_-incorporation into the aryl ring is through a single-oxygen insertion rather than the insertion of both oxygen atoms of the O_2_ as proposed earlier [33,55,58,61,62,63,64]. This incorporation of a single oxygen atom from O_2_ is analogous to the reaction of oxygenases in biological systems (Scheme 9c), which leads to distinct intradiol or extradiol ring-opening products, respectively, depending on the initial sites of O_2_ coordination [66,67,68,69]. It is expected that the activation and insertion of O_2_ into aromatic ring in the present case is dependent on its coordination to the Ti–sites on the TiO_2_ surface, just as in the active centre of oxygenases.

**Scheme 10:**
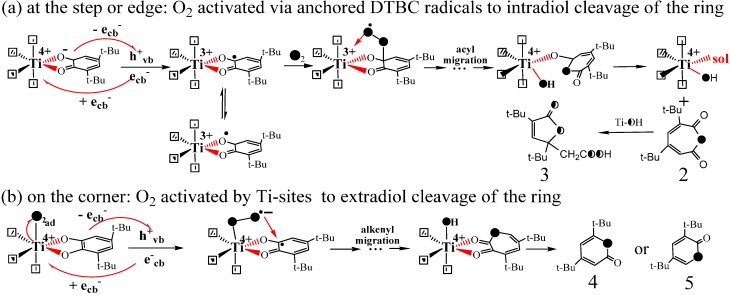
Proposed mechanism for singly O-atom incorporation in the photocatalytic cleavage of catechol by TiO_2_. (**a**) intradiol cleavage via the anchored DTBC radicals active dioxygen at the step or edge, (**b**) extradiol cleavage via Ti-sites active dioxygen on the corner [53].

Also interestingly, it was found that the different TiO_2_ particles with identical crystal structures and similar defect concentrations in bulk, but different exposed Ti-site coordination on the surface exhibited different the distribution in the intradiol products (**2** and **3**) and extradiol products (**4** and **5**) [53]. The ratio of intradiol to extradiol products had a good correlation with the ratio of Ti-**4c** to Ti-**3c** sites. Since the relative amounts of the exposed Ti-**4c** to Ti-**3c** sites would be largely dependent on the TiO_2_ particle size, the ratio can be determined by the inherent geometric size of the particles. The most noteworthy fact was that even for the smallest size of TiO_2_ particles (~9.7 nm), the formation of intradiol products still exceeds that of extradiol products. This was consistent with the geometrical proportions of Ti-**4c** and Ti-**3c** sites on the surface of any size of TiO_2_ nanocrystals. Thus, it was proposed that particle size determines the surface Ti-site coordination state, and then determines the chemoselectivity via the different activation pathways of O_2_ [53]. The high proportion of the intradiol products must be yielded on the steps (Ti-**4c**) or kinks, where the O_2_ should be activated and incorporated by the anchored DTBC radicals since there is no available Ti-site left for O_2_ coordination and reduction (Scheme 10a). Similarly, a small proportion of the extradiol products should be delivered from the corners (Ti-**3c**) or partial oxygen vacancies (also Ti-**3c**) with the smallest distribution proportion on the surface, and the activation of O_2_ was performed by Ti^3+^ (or e_cb_^−^) because there is an available Ti-site left for O_2_ coordination (Scheme 10b). Finally, the Ti center channels the decomposition of these proxy adducts and leads to the ring-opening products by inducing the cleavage of O-O bond (Scheme 10). The mechanism suggests that it is the molecular oxygen that breaks the C-C bond of the aromatics. The surface-mediated aromatic ring cleavage mechanism appears in TiO_2_ photocatalytic system shows the site coordination, steric hindrance and stereoelectronic effects play more important roles than the single oxidation potential. It also provides further understanding of the essence of heterogeneous photocatalytic oxidation, namely the final conduction band electron or Ti-sites activating dioxygen.

## 4. TiO_2_ Photocatalytic Decarboxylation of Carboxylic Acids

**Scheme 11:**
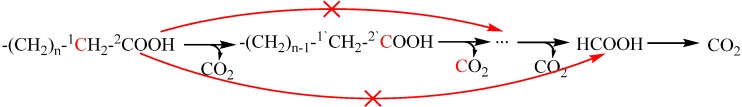
Stepwise cleavages of C^1^-C^2^ bonds in carboxylic acids.

As discussed above, the observed initial ring-opening products of aromatic compounds in aerated aqueous solutions are always carboxylic acids or carboxylic acid derivatives. Thus decarboxylation of these carboxylic acids is one of the most important steps for complete mineralization of organic pollutants. The most accepted mechanism for decarboxylation is through the Photo-Koble reactions [70], which is initiated by the hole oxidation of the carboxylic acids to release the CO_2_ and alkyl radicals. In the presence of O_2_, the alkyl radicals react with O_2_ to form the peroxyl radicals, and then decompose to hydroxylated and carbonylated intermediates via quaternion peroxide intermediates (Russell mechanism) [71]. However, the experimental results for the degradation of saturated monocarboxylic acids (from acetic acid (C_2_) to valeric (C_5_)) in aerated aqueous solution by TiO_2_ photocatalysis indicated that the photocatalytic oxidation of the acids led to the release of CO_2_ and the formation of carboxylate acid with one less carbon, that is, a C_5_ acid sequentially formed C_4_ products, then C_3_ and so forth (Scheme 11) [44,72], but little hydroxylated and carbonylated intermediates were detected. This means that there are other possible mechanisms for the TiO_2_ photocatalytic decarboxylation of carboxylic acids other than the Photo-Koble process and Russell mechanism.

Since this decarboxylation breaks the C^1^-C^2^ bond of the original acid and establishes a new carboxyl group in the immediately smaller acid, it is feasible to directly trace these processes by using the 18-oxygen isotopic labeling method. The photocatalytic decarboxylation of propionic acid was carried out in H_2_^18^O solution to observe the ^18^O profile of the produced acetic acid [44]. The isotope-labeling results showed that both the oxygen atoms of O_2_ and H_2_O can incorporate into the acetic acid. The percentage of the O_2_-incorporation can reach as much as 42% at a conversion of 25.1%. To obtain information about the intermediates generated during decarboxylation, diffuse reflectance FTIR measurements (DRIFTS) were employed to monitor *in-situ* the oxidative decarboxylation process of propionic acid. During irradiation, an absorption peak of C=O stretch of α-keto group of pyruvic acid was observed. This peak shifted from 1772 cm^−1^ to 1726 cm^−1^ when the 18-oxygen isotope labeling of H_2_^18^O was introduced, indicating that pyruvic acid is the intermediate of decarboxylation of propionic acid, and the oxygen atom of α-keto group comes from H_2_O (Equation (6)) [44]:

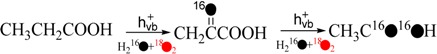
(6)

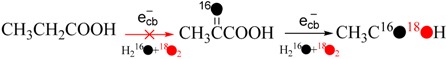
(7)

**Scheme 12:**
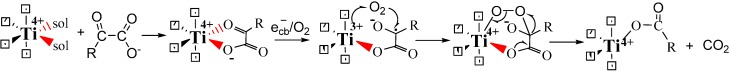
A possible pre-coordination mechanism for dioxygen incorporation into the product during the decarboxylation of α-keto acids [44].

The isotope labeling results on the decarboxylation of propionic acid showed the O atom in O_2_ was incorporated into the product acetic acid, while O_2_ did not participate into the formation of the intermediate pyruvic acid (Equation (7)) [44]. Thus O_2_ should be introduced during the oxidation of pyruvic acid to acetic acid. By using pyruvic acid as the model substrate, the 18-oxygen labeling experiment in ^18^O_2_/ H_2_^16^O was carried out to examine the transformation of pyruvic acid to acetic acid by TiO_2_ photocatalysis. The oxygen atom of O_2_ was largely incorporated into the acetic acid, and at least one oxygen atom of the substrate pyruvic acid was preserved in the carboxyl group of the formed acetic acid. The proportion of O_2_-incorporation greatly depended on the reaction conditions, because the O_2_-involved decarboxylation competes with the hole/OH radical-promoted decarboxylation process. The electrochemical experiment further confirmed that, in the absence of holes/OH radicals, O_2_ could independently cleave the C^1^-C^2^ bond of pyruvic acid to generate acetic acid with 100% selectivity at a negative bias, while no reaction was observed in the case of propionic acid [44]. A reasonable explanation for such a difference is the different modes of coordination of propionic acid and pyruvic acid on the TiO_2_ surface. An α-keto acid adsorbs on the TiO_2_ surface via bidentate coordination with the Ti sites, which is favorable for the incorporation of O_2_ into the C^1^-C^2^ bond, likely through a Crigee rearrangement (Scheme 12). However, for propionic acids, it can only chemisorb via monodentate coordination which is not favorable for O_2_-incorporation.

**Figure 2:**
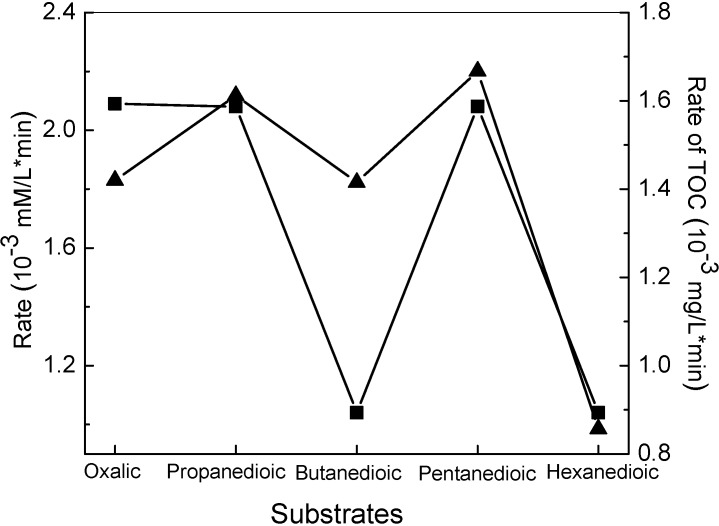
Average rates for both full conversion (■) and TOC removal (▲) by TiO2-based photocatalysis for the five dicarboxylic acids as a function of their carbon number [43].

For the decarboxylation of dicarboxylic acids, the process was also found to proceed through stepwise loss fo carbon atoms [43]. More interestingly, the dicarboxylic acids with an even number of carbon atoms (e-DAs) always degraded more slowly than those acids with an odd number of carbon atoms (o-DAs) (Figure 2).

**Scheme 13:**
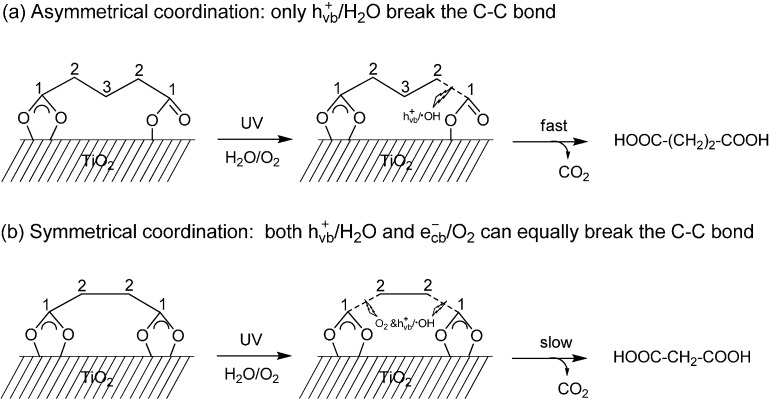
Schematic diagrams of photoctalytic decarboxylation of dicarboxylic acids. (**a**) Pentanedioic acid represents dicarboxylic acids with an odd number of carbon atoms (o-DAs); (**b**) butanedioic acid represents dicarboxylic acids with an even number of carbon atoms (e-DAs) [43].

The attenuated total reflection FTIR (ATR-FTIR) combined with the ^13^C labeling method showed that both carboxyl groups of the e-DAs acids coordinate to TiO_2_ through bidentate chelating forms. However, for the o-DAs acids, only one carboxyl group coordinates to TiO_2_ in a bidentate chelating manner and the other carboxyl group interacts with the surface in a monodentate mode. Further 18-oxygen labeling experiments showed that the photocatalytic oxidation of o-DAs and e-DAs had different oxygen sources in the carboxyl group of the formed decarboxylated products of dicarboxylic acid [43]. For o-DAs, which have two adsorption different modes: the asymmetrical bidentate and monodentate chelating mode, only the oxygen from H_2_O was incorporated into the initial decarboxylated products (Scheme 13a). In contrast, for the e-DAs, which exhibit symmetrical bidentate chelating mode on TiO_2_ surface both O_2_ and H_2_O contributed to their decarboxylated products (Scheme 13b). All these results indicated that the coordination patterns of the substrates on the TiO_2_ surface are very important in the TiO_2_ photocatalytic decarboxylation of saturated moncarboxylic or dicarboxylic acids and are sometimes the main factor that determines the active species, such as h^+^_vb_/∙OH and e^−^_cb_/O_2_, to cleave the C-C or C-H bond of the substrates.

## 5. Conclusions

In this review, we have summarized our recent work on the oxygen-18 labeling method to unravel the TiO_2_-photocatalytic mechanisms, focusing mainly on the mechanisms of hydroxylation of aromatics, cleavage of aryl rings and decarboxylation of saturated carboxylic acids. The incorporation of oxygen atoms from O_2_ or H_2_O into the initial products is tracked using oxygen-18 labeled ^18^O_2_ or H_2_^18^O in these oxidation processes and could help us understand the essential mechanisms of TiO_2_ photocatalytic oxidation. The isotope results indicated that the mineralization of organic compounds needs the cooperation of H_2_O/h^+^_vb_ and O_2_/e^−^_cb_ in the TiO_2_ photocatalytic oxidation. The activation and incorporation of molecular oxygen which is the final oxidant of TiO_2_ photocatalytic oxidation are mainly affected by the coordination environment of Ti sites on the TiO_2_ surface. This site coordination plays a more important role than the single oxidation potential in the photocleavage of aromatic rings. It also showed that ^18^O-isotope labeling is a very efficient and powerful method for unraveling the TiO_2_-photocatalytic mechanisms which are the basis of incorporation of oxygen into products. However, one must be mindful of the processes of oxygen isotopic exchange and photoinduced oxygen isotopic exchange when using the oxygen-18 labeling method in TiO_2_-photocatalysis systems
